# Effective and Efficient Correction of Severe Skeletal Class II Division 1 Malocclusion with Intermaxillary Elastics

**DOI:** 10.1155/2021/6663563

**Published:** 2021-03-02

**Authors:** Mohammed K. Badri

**Affiliations:** Department of Pedodontics & Orthodontics, College of Dentistry, Taibah University, Madinah, Saudi Arabia

## Abstract

Treatment of Class II malocclusion accompanied with a skeletal discrepancy is challenging. The approach of correction depends on several factors such as the status and pattern of growth, severity of the malocclusion, and patient cooperation. This case report describes a successful management of a 12-year-old young adolescent boy that was presented with a Class II division 1 malocclusion with an underlying skeletal discrepancy in horizontal and vertical dimensions. Growth modification was achieved by means of bite opening and unlocking the mandible together with Class II elastics and mechanics. Treatment was highly effective and efficient by achieving all treatment goals within a period of 18 months.

## 1. Introduction

Class II dental malocclusion considered as the second most prevalent malocclusion after Class I, with a prevalence range of 13%-24% [1–5]. As described and categorized by Edward Angle more than a century ago, Class II division 1 malocclusion is characterized by distal occlusion of the lower first molars by at least a half cusp width in relation to upper first molars resulting in locking of the mandible in a distal position. Moreover, specific traits of this division were the protrusion of the upper incisors and interruption of the relation of upper and lower incisors by the lower lip [6, 7]. Nevertheless, Class II malocclusion often accompanied by a skeletal discrepancy that could be caused by either a deficient mandible, excessive growth of maxilla, or a combination of both [8, 9].

Treatment approach and modality for Class II division 1 with an underlying skeletal problem depend on several factors that should be considered by the clinicians such as chronological age, growth potential, skeletal maturity, severity of the condition, and patient motivation and cooperation [10, 11]. The treatment options for growing patients with such malocclusion mainly based on growth modification on an exact time-frame taking advantage of the adolescent growth spurt. Failure to initiate proper treatment within the proper timing will lead to deviation of treatment option towards camouflage or even orthognathic surgery. Treating growing adolescents could involve the use of Class II elastics, headgear, or functional appliances in order to restrain further maxillary growth and promote favorable mandibular growth.

Several studies showed that over the long-term treatment results achieved with Class II elastics are similar or comparable to those achieved with functional appliances, given that treatment started at an appropriate timing [12–17]. Both Class II elastics and functional appliances contributed to correction of the malocclusion by skeletal and dental changes, but Class II elastics resulted in a more dentoalveolar modification [17–19]. Several dentofacial changes are associated with the use of Class II elastics such as maxillary retraction, mandibular protraction, increase of lower anterior facial height, clockwise rotation of the occlusal plane, retroclination of maxillary incisors, proclination of mandibular incisors, and forward movement and extrusion of mandibular molars [16–22]. Most of the effects of Class II elastics are favorable, but in order to control any unwanted side effects, a good case selection and application of appropriate biomechanics should be considered by the orthodontist.

In the current case report, we discuss the treatment of a Class II division 1 malocclusion accompanied with a severe Class II skeletal discrepancy and hypodivergent growth pattern in a growing adolescent.

## 2. Diagnosis and Etiology

**Figure 1:**
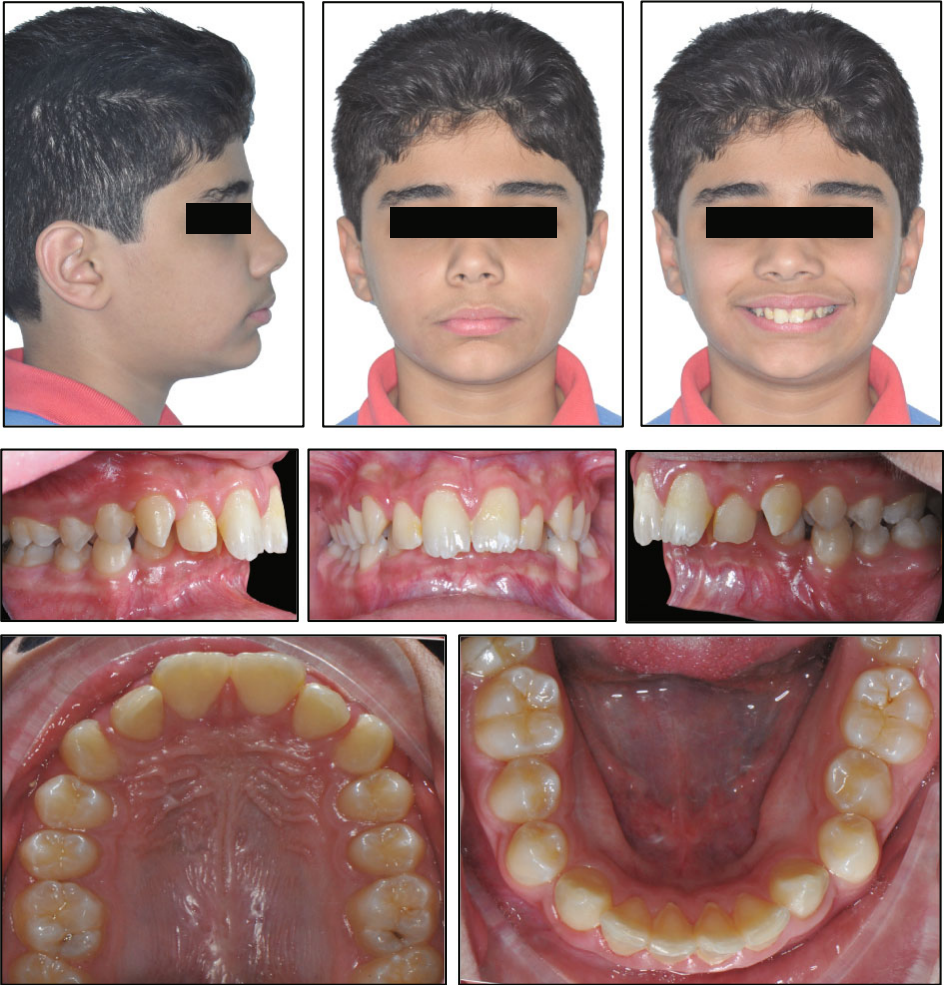
Pretreatment facial and intraoral photographs.

12 years and 8 months young boy referred to the orthodontic specialty clinic with a chief complaint of “protruded front teeth”. Medical and dental history indicated no significant findings. Extraoral clinical examination revealed a symmetrical euryprosopic face type, convex soft tissue profile, retruded mandible, deep mentolabial sulcus, decreased lower facial third, and on smiling lower lip was trapped by the upper central incisors (Figure 1).

**Figure 2:**
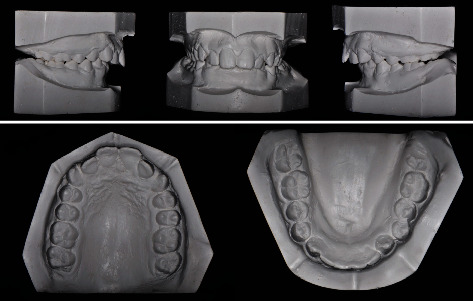
Pretreatment orthodontic study models.

**Figure 3:**
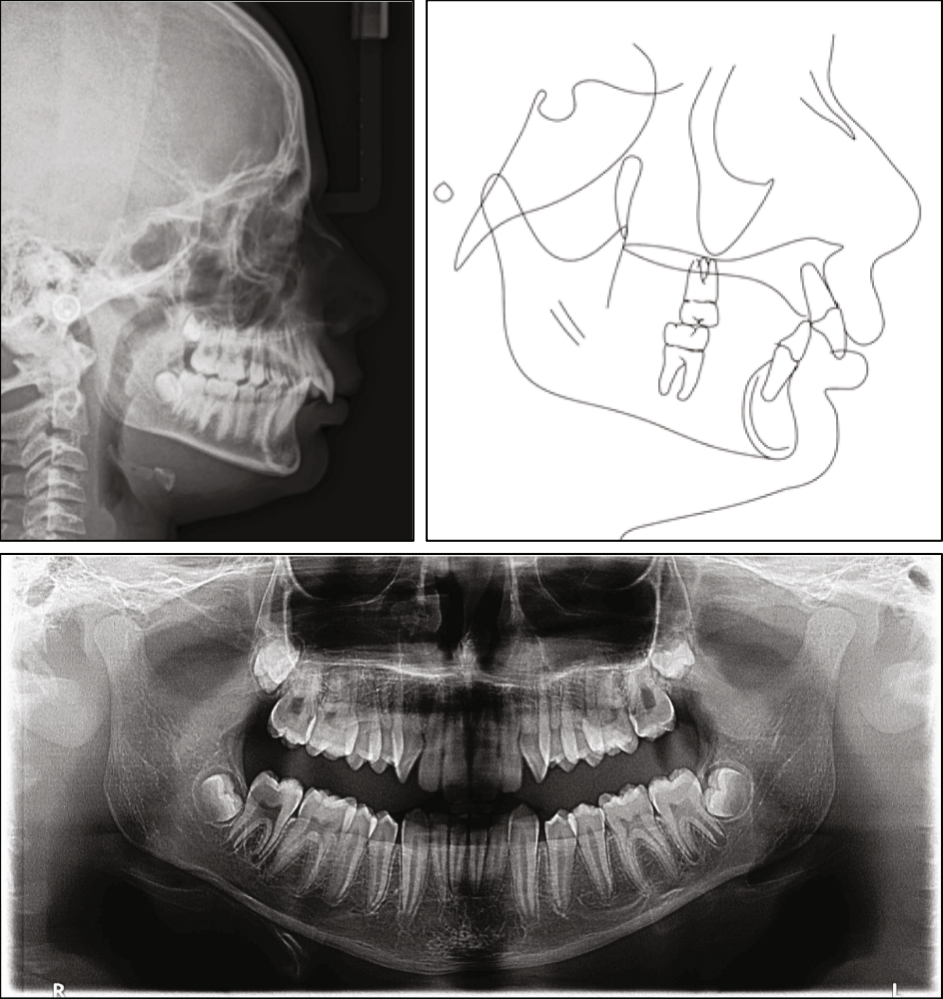
Pretreatment radiographs and tracing.

Intraoral and dental cast examination showed Class II division 1 malocclusion with full-step molars and canines bilaterally, severe overjet of 9 mm, overerupted lower incisors with deep (100%) impinging overbite, deep curve of Spee (COS) of 3 mm, and unilateral posterior cross-bite on the upper left 1^st^ and 2^nd^ molars. Mild anterior crowding with rotated canines (uppers rotated disto-palatal and lowers rotated mesio-lingual). Patient had poor oral hygiene, plaque accumulation, and mild gingival inflammation. All permanent teeth are erupted while the 3^rd^ molars are still developing (Figures 1–3).

Cephalometric analysis revealed a severe skeletal Class II relationship (ANB = 7.8°) with horizontal growth pattern tendency (hypodivergent mandibular plane angle, MPA = 28.1°, FMA = 19.6°), and anterior facial height is markedly reduced. Cervical vertebral maturation (CVM) indicates cervical stage 1 (CS1). Lower incisors are proclined (L1 − MP = 99.3°), and both lips are anterior to the E-line by 1 mm (Figure 3 and Table 1).

## 3. Treatment Objectives

The following were the list of treatment objectives: (1) improve the sagittal and vertical skeletal relationships; (2) enhance and guide mandibular growth; (3) resolve increased overjet; (4) establish Class I canine and molar relationship; (5) resolve the impinging overbite, deep COS, and traumatic occlusion; (6) resolve the left posterior cross bite; (7) resolve crowding and rotations; and (8) improve facial profile and smile esthetics.

## 4. Treatment Alternatives

Given the patient's age and problem list, a nonsurgical and nonextraction treatment plan based on growth modification to enhance mandibular growth was developed. The primary plan presented to the patient and his family was to start with growth modification using a removable Class II functional appliance or cervical pull headgear with anterior bite plane followed by leveling and aligning with a fixed edgewise appliance. The option was not considered by the family due to increased cost from the use of additional device. In addition, the treatment duration could be prolonged especially if it was divided into two phases. Therefore, an alternative plan addressing those concerns was presented which involved bonding a fixed edgewise appliance, anterior bite turbo, and simultaneous growth modification using intermaxillary elastics taking advantage of the patient's early adolescence and prepubertal stage of development.

## 5. Treatment Progress

The patient demonstrated a poor oral hygiene and few carious lesions, so before starting orthodontic treatment, oral hygiene instructions were given, and patient was referred for periodontal and restorative clearance.

Once cleared, a full-fixed straight wire appliance of slot size 0.022^″^ × 0.028^″^ MBT was bonded in both arches. Leveling and aligning lasted for 6 months, which were achieved by the following NiTi wire sequence (0.014^″^, 0.016^″^, 0.018^″^, 0.016^″^ × 0.022^″^) then followed by stainless steel (SS) working wires (0.016^″^ × 0.022^″^, 0.018^″^ × 0.025^″^, and 0.019^″^ × 0.025^″^). SS arch wire expansion and criss-cross elastic from palatal of upper left first molar to buccal of lower left molar were used to correct the unilateral posterior cross bite on left molars.

**Figure 4:**
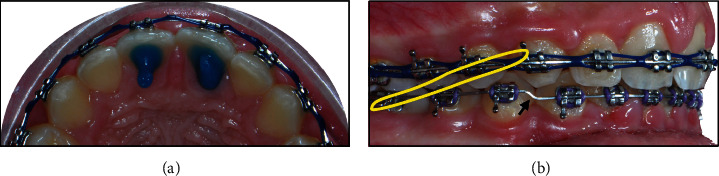
(a) Anterior bite turbo cemented on palatal of maxillary central incisors. (b) The use of reverse COS in the lower arch wire, step down bends (black arrow) for lower anterior teeth, and Class II elastic bands (yellow imaginary band).

In addition, to address the vertical and sagittal skeletal problems, anterior bite turbos palatal to upper central incisors were cemented (Figure 4(a)); gradual step-down of lower anterior teeth (total of 3 mm) and reverse curve of Spee were implemented by the lower SS arch wires (Figure 4(b)). Moreover, Class II intermaxillary elastics were started using light forces then replaced by medium force elastics as we progressed in treatment (Figure 4(b)).

After 10 months in treatment, the antro-posterior canines and molars relationships were corrected. Final detailing and finishing included arch wire adjustments and power chain use for some remaining spaces closure. The fixed appliance was debonded after 18 months of active treatment. Clear vacuum retainers were delivered, and after 1 month, they were replaced by Hawley retainers with anterior bite plate on the upper appliance.

## 6. Treatment Results

**Figure 5:**
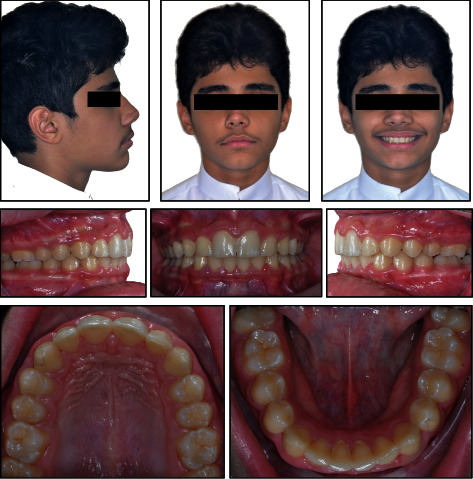
Posttreatment facial and intraoral photographs.

**Figure 6:**
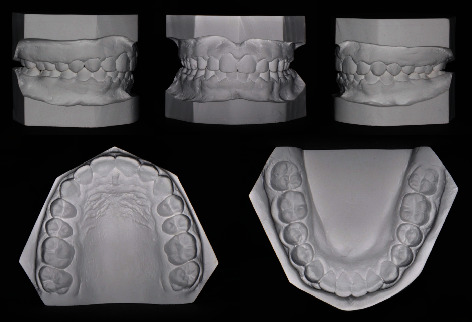
Posttreatment orthodontic study models.

The overall outcome of treatment was successful, and the patient's chief complaint was addressed. Treatment objectives were achieved in an efficient treatment duration (18 months and 14 visits). The skeletal and dental relationships were significantly improved. Facial profile showed great improvement as facial proportions were restored, mandible was advanced, convexity was reduced, and restored normal mentolabial sulcus. Moreover, smile esthetics were improved, and lip position was corrected with no entrapment (Figure 5). Intraoral photographs and dental models showed that Class I canine and molar were established both sides, normal overjet and overbite were achieved, and correction of deep COS and overerupted lower incisors. The unilateral posterior cross bite on left side molars was corrected with resulted increase in intermolar width of upper arch (Figures 5 and 6).

**Figure 7:**
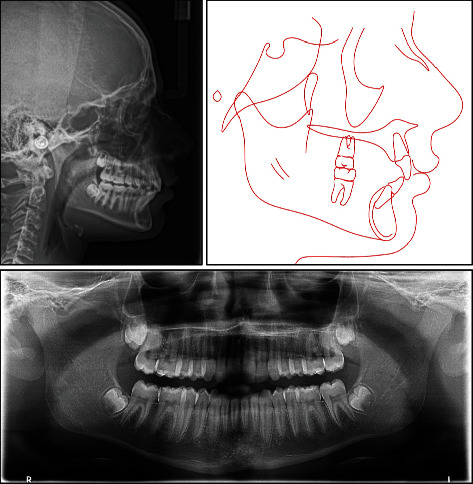
Posttreatment radiographs and tracing.

**Figure 8:**
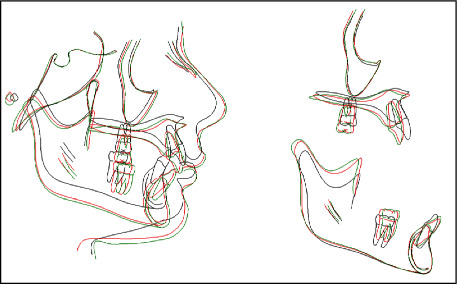
Superimposition of pretreatment (black), posttreatment (red), and follow up (green) lateral cephalograms.

Lateral cephalometric analysis and superimposition showed significant skeletal improvement in sagittal and vertical dimensions with clockwise rotation of the mandible. Antro-posterior skeletal relationship changed from pretreatment (ANB 7.8°, Wits 5.6 mm) to posttreatment (ANB 4.8°, Wits 0.4 mm), facial convexity decreased from 12.9° to 6.9°, mandibular plane angle increased from (SN-MP 28.1°, FMA 19.6°) to (SN-MP 31°, FMA 21.4°), total face height (Na-Gn) increased from 105.2 mm to 116.9 mm, lower face height (ANS-Gn) increased from 59.7 mm to 65.5 mm, and the hypodivergent growth pattern improved from (*y*-axis 64.7°) to (*y*-axis 68.1°). Maxillary incisors were retroclined, and the deep bite was corrected by relative intrusion of a combination of molar extrusion and mandibular incisors intrusion and proclination. In addition, mandibular molars moved mesially while maxillary molars slightly distalized (Figures 7 and 8, Table 1).

**Figure 9:**
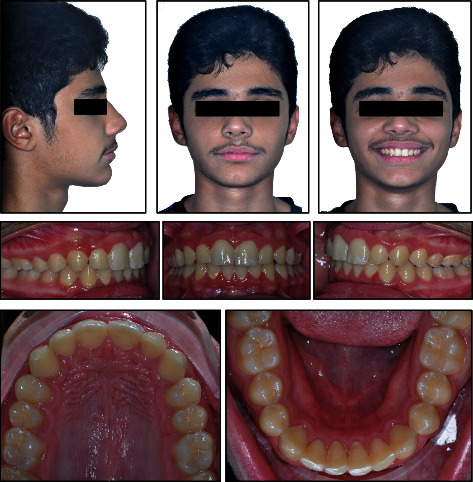
1-year follow-up facial and intraoral photographs.

**Figure 10:**
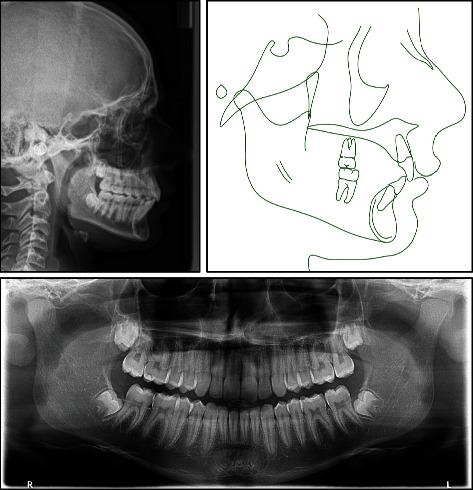
1-year follow-up radiographs and tracing.

At 1-year follow-up, the occlusion was stable with no significant relapse, and the patient demonstrated poor oral hygiene with mild gingival inflammation (Figure 9). Lateral cephalometric superimposition showed that both maxilla and mandible still growing in a favorable downward and forward direction and sagittal and vertical relations are maintained. Maxillary incisors slightly proclined while mandibular incisors slightly extruded, and its inclination to mandibular plane was improved (Figures 8 and 10, Table 1).

## 7. Discussion

This case report showed a successful management of a 12-year-old adolescent boy presented with a Class II division 1 malocclusion accompanied with a severe skeletal Class II discrepancy. Several key factors contributed to the success of this treatment mainly including early intervention at prepubertal stage, pattern of skeletal growth, patient's cooperation, and mechanics applied.

The time of intervention in this case was of great impact in correcting the skeletal discrepancy. The treatment started when the patient was in the prepubertal stage of growth. The patient was at early stage of CVM (CS1, Figure 3), secondary sexual characteristics did not appear, and no significant increase in patient's height during the last 2 years, indicating that the growth spurt was yet to be hit. At posttreatment time point, the patient was in the postpubertal stage of maturity marked by the CVM (early CS4, Figure 7), appearance of secondary sexual characteristics (such as facial hair and hoarse voice), and noticeable increase in height, thus indicating that growth spurt had hit during treatment. At the 1-year follow-up with a CVM of early CS5 (Figure 10), patient showed further mandibular growth with an increase of 2 mm in length; however, the mandibular growth spurt occurred during treatment with an increase of 6.7 mm (Co-Gn at pretreatment = 111.3 mm, Co-Gn at posttreatment = 118 mm, Co-Gn at follow − up = 120 mm). Cases of severe Class II skeletal problems are better to be treated as early as possible and to take advantage of the patient's growth spurt [9, 15]. Moreover, regardless the type of intervention, the earlier the treatment, the greater the impact on mandibular growth [23] and the more stable the results are on the long term [15].

In the current case, upon opening the patient's bite and releasing the locked mandible from the impinging overbite, a great improvement in sagittal skeletal relation was achieved. Angle indicated that in Class II malocclusion, the lower dentition is locked in distal occlusion, and thereby, the mandible will be locked as well [6]. Several studies advocated that dental and occlusal interferences will restrict normal range of movement of the mandible by displacing it in a more posterior position and will hinder its normal growth [24–26]. Removal of such interferences by opening the bite will allow the mandible to shift forward, establish normal range of movement, reposition the condyles in the center of the fossae, and promote mandibular growth, thus reducing the sagittal skeletal discrepancy [9, 24–28]. In a study of 60 patients with Class II deep bite of hypodivergent and normodivergent vertical relations, they showed great forward movement of the mandible with normal growth after bite opening [28].

It was well noticed that most of the sagittal discrepancy was improved after removing the restriction from the impinging deep bite and unlocking the mandible, while the use of Class II intermaxillary elastics was for a short period still effective to add to the final sagittal correction. Class II elastics carried out further guidance and enhancement of mandibular growth toward normal skeletal relation, added considerable restriction to the growth of the maxilla, and improved the vertical skeletal relationship (Table 1). Despite the correction of the sagittal skeletal problem and the increase in the mandibular body and ramus size, the patient did not show increase in the SNB angle (Table 1). This could be explained by the backward rotation of the occlusal plane and mandible from Class II elastics and mechanics used [18, 20, 22]. In hypodivergent growing patients, bite opening is achieved by incisors intrusion, molars eruption, or a combination of both. If the amount of molar extrusion overcame the amount of vertical condylar growth, a backward mandibular rotation will occur [27]. Therefore, a better reflection of the sagittal results will be using the Wits appraisal [29], and in this study, Wits improved significantly from 5.4 mm at pretreatment to 0.4 mm at posttreatment indicating Class I skeletal relationship (Table 1).

The vertical skeletal relationship improved significantly from the correction of the deep bite by use of reverse COS wires, Class II elastics, and bite turbos. Those mechanics helped to extrude the molars, intrude and procline the incisors, and facilitate further mandibular growth. As mentioned earlier, with Class II elastics, there is a clockwise rotation of the occlusal plane and mandible with a final outcome of increase in lower anterior face height [18, 20, 22]. Such changes considered to be highly favorable in the current case which helped improve the hypodivergent vertical relationship.

The skeletal growth pattern of the patient was a favorable adjunct in the management of his case and helped in accommodating the current treatment plan. The vertically hypodivergent mandibular plane angle, the forward rotation of the mandible, and the underdeveloped anterior face height facilitated in toleration of the applied extrusive mechanics and the resulted backward rotation of the mandible. On the opposite, cases of vertical growth pattern with high mandibular plane angle are challenging and difficult to treat, and applying such extrusive mechanics should be avoided as they would deteriorate the final results and will not work in favor of the patients while more control of the vertical dimension should be considered [30].

Among the challenges that were faced in this case was to maximize the orthopedic changes using Class II elastics while minimizing elastics possible side effects. Proclination of lower incisors was significantly increased (Table 1) from Class II elastics, reserve wires use, and correction of deep COS. Such side effect could have been minimized if strict anchorage preparation was applied. Tweed advocated for rigorous anchorage preparation prior to the application of Class II elastics and mechanics in order to avoid proclination of anterior teeth [31]. In addition, Class II skeletal cases with excellent anchorage preparation prior to treatment showed greater orthopedic effects (such as maxillary retraction and mandibular protraction) comparing to cases treated with no anchorage preparation [21].

## 8. Conclusion

Class II elastics considered a viable orthodontic option for correcting Class II skeletal discrepancy in growing patients, given the careful case selection based on timing of growth spurt, pattern of skeletal development, severity of the malocclusion, and patient's cooperation and motivation.

Moreover, orthodontists should consider the benefits and potential side effects of Class II elastics use, plan that in the treatment, and be able to handle and direct them to the patient's favor once executed in treatment.

## Figures and Tables

**Table 1:** Cephalometric measurements.

Measurement	Pretreatment	Posttreatment	1-year follow-up	Norm
Skeletal (sagittal)				
SNA (°)	85.8	81.5	82	82 ± 3.5
SNB (°)	78	76.7	77.5	80 ± 3.5
ANB (°)	7.8	4.8	4.5	2 ± 2
Wits (mm)	5.6	0.4	1	5
Convexity (NA-APg) (°)	12.9	6.9	6.2	0 ± 5
Skeletal (vertical)				
SN-MP (°)	28.1	31	30.8	32 ± 5
FMA (°)	19.6	21.4	21.2	24 ± 4.5
*Y*-axis (SGn/SN) (°)	64.7	68.1	68	67 ± 5.5
Dental				
U1-SN (°)	101.5	93.6	96.2	103 ± 5.5
U1-NA (mm)	2.8	0.7	2	4.3 ± 2.7
Interincisal angle (°)	132	123.6	127.6	130 ± 6
L1-MP (°)	99.3	111.5	105.8	95 ± 7
L1-NB (mm)	4.1	5.6	5.5	4 ± 1.8
Soft tissue				
Nasolabial angle (°)	118.2	111.7	116.6	102 ± 8
Upper lip to E-line (mm)	0.8	-1.8	-1.2	−4 ± 2
Lower lip to E-line (mm)	1.3	-1.2	-2.4	−2 ± 2
